# Three-Dimensional Globe Repositioning Following Orbital Reconstruction Independent of Bony Landmarks and Fracture Pattern Associations

**DOI:** 10.3390/cmtr19020025

**Published:** 2026-05-21

**Authors:** Aaron De Poortere, Kathia Dubron, Justine Neyt, Reinhilde Jacobs, Eman Shaheen, Robin Willaert

**Affiliations:** 1 Department of Oral and Maxillofacial Surgery, University Hospitals Leuven, 3000 Leuven, Belgium; 2 OMFS IMPATH Research Group, Department of Imaging & Pathology, University Hospitals Leuven, 3000 Leuven, Belgium; eman.shaheen@uzleuven.be; 3 Department of Dental Medicine, Karolinska Institutet, 171 77 Stockholm, Sweden

**Keywords:** orbital trauma, orbital floor fracture, globe position, globe repositioning, dystopia, enophthalmos

## Abstract

Orbital fractures account for up to 16% of all facial fractures. Surgical intervention for these fractures depends on several factors, including globe displacement. This case-control study aimed to quantify the effects of orbital reconstruction on the three-dimensional globe position after orbital trauma. Pre- and post-operative radiological data of 29 cases (27 patients, with two patients requiring reintervention) with an orbital fracture were analyzed. The contralateral, unaffected orbits of the same patients served as the control group. This study used a recently validated method employing semi-automatic registration algorithms to measure globe displacement after trauma and its post-operative change. The statistical analysis, performed using the Mann–Whitney U test, showed significant globe position restoration in the anterior–posterior (2.15 mm, *p* ≤ 0.001) and medial–lateral (1.34 mm, *p* ≤ 0.001) directions, as well as in overall three-dimensional Euclidean distance (3.04 mm, *p* = 0.014) after surgery. The repositioning was clinically relevant in 62% of the cases, indicating that the three-dimensional globe repositioning was 2 mm or more with a positive clinical impact. Additionally, it was important to note that both the number of fractured walls and fractures of the inferomedial strut significantly affected globe repositioning after trauma. This finding highlights the importance of accurately considering the fracture pattern in surgical planning, as a better understanding of globe displacement following orbital trauma could enhance patient selection, surgical planning, and clinical outcomes.

## 1. Introduction

Orbital fractures are a common type of injury, representing up to 16% of facial fractures [1,2]. They usually involve displacement of the orbital floor and/or medial wall. The decision to perform surgery depends on several factors, including fracture pattern, clinical symptoms, and the patient’s profile [1,3]. While acute muscle entrapment is the only absolute indication for urgent intervention, relative indications include persistent diplopia, extraocular motility dysfunction, and globe displacement greater than 2 mm [1,3,4]. The late-onset or spontaneous resolution of these symptoms makes the decision for surgery even more challenging [5]. In the literature, the effect of different fracture patterns on enophthalmos has been discussed, but no clear agreement has been reached on their predictive value for enophthalmos [6]. One relevant anatomical structure is the inferomedial strut. This thin, bony junction between the medial orbital wall and floor is an important anatomical structure that supports the globe, and its angle is usually around 110° [7]. In other pathologies, such as in patients with Graves’ disease, this landmark has been thoroughly investigated in orbital decompression surgery [8]. Therefore, this anatomical landmark was investigated in the present study in the context of orbital trauma, with particular attention to the fracture pattern, to assess its role in globe positioning.

Orbital fractures can cause globe displacement, clinically presenting as enophthalmos or hypoglobus, and lead to functional problems (such as diplopia) and cosmetic concerns [3,6]. Accurate assessment of globe position is, therefore, important during pre-operative evaluation, surgical planning, and outcome evaluation. Clinically, this can be assessed through a physical examination. Patients with enophthalmos may present with a smaller, more recessed eye, a narrower palpebral fissure, and supra-orbital hollowing or shadowing [9]. Objective measurements for enophthalmos are typically obtained using an exophthalmometer, or can be assessed using radiological techniques [10,11,12]. The problem is that both techniques rely on intact bony landmarks to establish a reference plane, thereby reducing accuracy in patients with multiple facial fractures. Furthermore, to evaluate globe displacement, most current techniques primarily focus on the axial plane [10]. However, this method underestimates globe displacement since orbital trauma affects all three dimensions. A three-dimensional (3D) assessment would provide a more accurate and comprehensive understanding, although its clinical value and correlation with post-operative outcomes are still not fully established [13,14,15].

Therefore, the aim of this study was to quantify the effects of orbital reconstruction on post-traumatic globe displacement using a recently validated 3D analysis that is independent of bony landmarks [13]. Additionally, the association between 3D globe repositioning and predefined clinical or anatomical subgroups, including type of reconstruction material, fracture pattern, and clinical dystopia, was investigated.

## 2. Material and Methods

This research was conducted in compliance with the ethical standards of the Research Committee (Ethical Review Board, University Hospitals of Leuven, reference number S65739). It adhered to Good Clinical Practice standards and the principles of the World Medical Association Declaration of Helsinki.

### 2.1. Patient Selection

This retrospective study included clinical and imaging data from 27 patients with orbital floor fractures. All patients underwent surgery at the Department of Oral and Maxillofacial Surgery, University Hospitals of Leuven, between January 2013 and December 2023. Only adults (≥18 years) with clear surgical indications (eye motility problems, enophthalmos, and hypoglobus) were included. In all patients, the unaffected contralateral orbit served as the control group. Exclusion criteria encompassed (1) patients with naso-orbitoethmoid, Le Fort II, Le Fort III, or panfacial fractures; (2) those with pre-existing or concurrent (traumatic) ophthalmologic disease (including canthotomy) and who had undergone previous ocular or periocular surgery; and (3) patients with CT scans exhibiting motion artefacts or slice thickness >2 mm. Characteristics of the case and control groups were considered homogeneous, given that each control corresponded to the same patient as the case. Predefined subgroups were established based on type of reconstruction material (Patient-Specific Implant (PSI) vs. other), fracture pattern (number of walls fractured: 1 vs. >1; inferomedial strut intact vs. fractured), and pre-operative clinical dystopia (yes vs. no). The data was anonymously extracted from patient records, including demographics, pre-operative symptoms, surgical details (such as timing and type of reconstruction material), and follow-up information.

### 2.2. Analysis of Three-Dimensional Globe Displacement and Globe Repositioning

**Figure 1:**
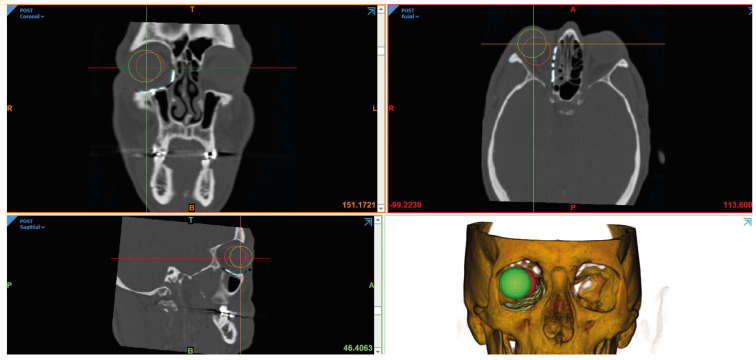
Defining globe position on pre- and post-operative CT. Globe position was defined by fitting a sphere to the globe’s circumference using Mimics Medical software (Materialise^®^, Leuven, Belgium). The pre-operative sphere is represented in red, while the post-operative sphere is shown in green. In this patient, an anterior–lateral repositioning following PSI reconstruction was observed.

A validated 3D CT method was used to measure globe displacement, demonstrating excellent intraclass correlation coefficient scores of ≥0.99 for both inter- and intra-observer testing [13]. All patients underwent pre- and post-operative CT scans of the maxillofacial skeleton, further described as “CT-pre” and “CT-post,” respectively. A single volume of data was acquired in the axial plane with a slice thickness of 0.75 to 2 mm and recorded in a Digital Imaging and Communications in Medicine (DICOM) format. The DICOM files were anonymized for further analysis. This method was used for both the affected orbit and the unaffected contralateral orbit. The following steps were performed:

1. Voxel-Based Registration: “CT-post” was registered to “CT-pre” using the skull base as a reference, employing Amira software (Version 6.7.0, ThermoFisher Scientific^®^, Merignac, France), resulting in a new DICOM dataset called “CT-postreg.”
2. Defining Globe Position: A sphere fitted to scleral landmarks was mapped onto the globe’s circumference in both the “CT-pre” and “CT-postreg” scans using Mimics Medical software (Version 21.0, Materialise^®^, Leuven, Belgium) (Figure 1).
3. Calculating Positional Changes: The changes between the centers of the spheres, representing the globes in “CT-pre” and “CT-postreg,” were calculated in the X, Y, and Z directions, along with the 3D Euclidean distance. The 3D Euclidean distance was used as a quantitative metric to assess spatial discrepancies between anatomical landmarks (mathematically, this is the straight-line distance between two corresponding points in 3D space) [16]. Each anatomical landmark was defined by 3D coordinates derived from CT imaging.

### 2.3. Statistical Analysis

Power calculations were performed using G*Power software version 3.1.9.6, assuming an alpha error probability of 0.05 and a power of 0.8 for the Mann–Whitney U test. This analysis indicated that a sample size of twelve patients would be needed to detect an effect size of 1.70, based on the mean and standard deviation from a previous validation study [13]. This means that the expected difference between the groups is 1.7 times the pooled standard deviation (large effect).

Statistical analysis was conducted using IBM SPSS Statistics^®^ (version 29.0.2.0). The Shapiro–Wilk test for normality was not significant (*p* > 0.05) for all three directions, nor for the 3D Euclidean distance in both the trauma and control groups. Therefore, changes in 3D were evaluated using the Mann–Whitney U test. The same testing was performed for the subgroup analysis. The *p*-values smaller than 0.05, with a 95% confidence interval, were considered statistically significant.

## 3. Results

A total of 27 patients (29 cases) diagnosed with orbital floor fractures were analyzed. The average age of the patients was 52.0 years (SD ± 17), with a male-to-female ratio of 2:1. Two of these patients underwent a secondary reconstruction, resulting in a total of 29 surgeries (or cases) analyzed. For all cases, the contralateral unaffected globe was evaluated using the same three-dimensional CT method and used as the control group (n = 29).

Accidental falls were identified as the most prevalent cause of injury, followed by assault, sports-related accidents, and traffic accidents (Table 1). Pre-operative clinical assessment (n = 27) revealed diplopia in 18 patients (67%) and hypoesthesia of the infraorbital nerve in 18 patients (67%). Enophthalmos was found in 12 cases (41%), and hypoglobus in four cases (14%). None of the cases showed acute inferior rectus muscle entrapment. Radiological assessment of the fracture pattern demonstrated, apart from orbital floor fractures, an associated fracture of the medial wall in 16 patients (59%) and the lateral wall in six patients (22%). The inferomedial strut, as stated above, was fractured in 14 patients (52%).

The indications for surgery for the cases were as follows: motility deficit (n = 15; 52%), extensive fractures (n = 12; 41%), and enophthalmos (n = 9; 31%). Enophthalmos was assessed clinically based on physician judgment and documentation (present/absent) in the medical records. Primary surgical reconstruction was performed at a median of five days post-trauma (range: 1–27 days; n = 19). As a tertiary referral center, some patients were referred for secondary reconstruction (n = 10). This group had a median interval of 132 days after the initial trauma. Various types of reconstruction materials were utilized: titanium mesh (DePuy Synthes^®^, Raynham, MA, USA or KLS Martin^®^, Tuttlingen, Germany) in 12 cases (41%), polydioxanone sheet in seven cases (24%), and PSI in 10 cases (34%). The mean follow-up duration was nine months (range: 1–25 months). In all but one patient, the post-operative CT was obtained within 24 h after surgery to minimize the effect of post-operative swelling. Eight patients were scanned intraoperatively.

**Figure 2:**
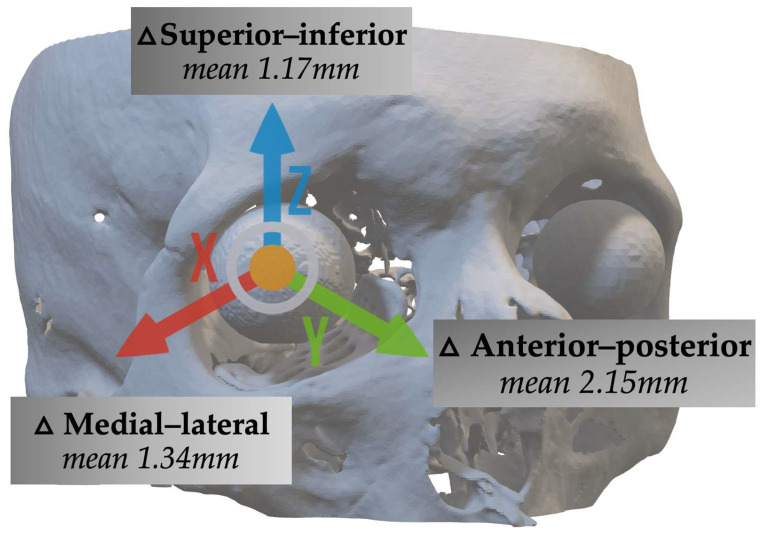
Assessment of globe repositioning after orbital reconstruction. Following orbital reconstruction, globe position shifted by 2.15 mm in the anterior–posterior direction, 1.34 mm in the medial–lateral direction, and 1.17 mm in the superior–inferior direction. The mean 3D Euclidean distance, representing overall globe repositioning, was 3.04 mm.

The globe position following orbital floor reconstruction demonstrated a mean repositioning of 2.15 mm, 1.34 mm, and 1.17 mm in the anterior–posterior, medial–lateral, and superior–inferior directions, respectively (Figure 2). The mean 3D Euclidean distance, representing the 3D shift in globe position, was 3.04 mm. An anterior–posterior shift of 2 mm or more was found in 13 cases (45%). A medial–lateral shift of 2 mm or more was found in seven cases (24%), and an inferior–superior shift of 2 mm or more was found in eight cases (28%). Seventeen cases (59%) had a globe shift of 2 mm or more in at least one direction. Overall, 3D repositioning of 2 mm or more was found in 18 cases (n = 62%). Globe replacement was less than 1 mm in all directions in eight cases (28%). Statistical analysis comparing the cases with the control group showed significant differences in the anterior–posterior (*p* ≤ 0.001) and medial–lateral (*p* ≤ 0.001) directions, as well as in the 3D Euclidean distance (*p* = 0.014). No significant difference was observed in the superior–inferior direction (*p* = 0.499), as detailed in Table 2.

In Table 3, firstly, subgroup analysis by reconstruction material shows that PSI use was associated with a higher mean Euclidean distance shift compared to other materials, with significant differences in the anterior–posterior and medial–lateral directions in both groups, and in overall Euclidean distance only for PSI.

Secondly, when stratified by fracture pattern, multi-wall fractures demonstrated greater globe shift than single-wall fractures. Significant differences were observed across anterior–posterior, medial–lateral, and Euclidean distance measurements in multi-wall fractures, whereas single-wall fractures showed significance only in the anterior–posterior direction. Similarly, inferomedial strut fractures were associated with increased globe shift compared to intact struts. Significant differences were observed in all directions, except in the inferior–superior direction, in the group with a fractured inferomedial strut, while no statistically significant differences were found in the intact group.

Finally, patients without pre-operative dystopia had a higher mean globe shift than those with dystopia. Significant differences were mainly observed in the anterior–posterior and medial–lateral directions in patients without dystopia, while in those with dystopia, significance was observed in the anterior–posterior, inferior–superior, and Euclidean distance measurements.

Overall, greater globe shifts were consistently associated with multi-wall fractures and inferomedial strut disruption.

## 4. Discussion

Accurate assessment of globe displacement following orbital trauma remains challenging due to the reliance on intact bony landmarks and the currently used two-dimensional axial measurements of globe displacement. These limitations are especially pronounced in complex fracture patterns, where anatomical disruption both impairs a reference plane and leads to underestimation of displacement of the globe in its three-dimensional space. In the present case-control study, these constraints were overcome by using a validated landmark-independent 3D approach, enabling a comprehensive evaluation of globe position, its association with clinical and anatomical variables, and the impact of various fracture patterns and reconstruction materials on the degree of position restoration. If a mirroring approach had been used, the analysis would rely on bilateral symmetry and on intact bony landmarks, which are frequently compromised in trauma, thereby introducing potential inaccuracies.

A significant correlation was identified between orbital floor reconstruction and globe repositioning, specifically in the anterior–posterior, medial–lateral, and 3D Euclidean distances. These findings suggest that orbital floor reconstruction effectively restores globe position following trauma, but also highlight its potential to induce considerable changes in globe position. Given that orbits are divergent, forward displacement is often associated with lateral displacement, as reflected in the significance of the medial–lateral dimension [3,9,17]. The lack of significant change in the superior–inferior direction aligns with the clinical observation that orbital floor trauma most commonly results in posterior displacement of the globe (enophthalmos) [3,18]. In this study population, the prevalence of enophthalmos was indeed 41%, whereas hypoglobus (inferior displacement) was observed in only 14%.

In a previously published study, healthy individuals demonstrated a normal variance of approximately 0.5 mm (±0.3 mm) for each axis and 1 mm (±0.4 mm) for the 3D Euclidean distance [13]. In the present study, similar results were seen in the control group. This suggests that minor changes in globe position following orbital floor reconstruction may go unnoticed. The existing literature generally states that a change of 1 cc in orbital volume induces a 1 mm globe shift [15,17]. However, since measuring orbital volume is not feasible in routine clinical practice, it remains unclear how much globe displacement is causing visible asymmetry. Clinically, enophthalmos becomes evident when there is a difference of 2 mm compared to the contralateral side [1,3,18,19]. Nevertheless, most patients tend to notice enophthalmos only when the discrepancy exceeds 3 mm [20]. If a globe position displacement of 2 mm (or more) is considered clinically relevant, in this study, 59% of the cases met this criterion in at least one 3D direction. Specifically, in the anterior–posterior dimension, 45% of the cases showed clinically relevant globe repositioning after surgery, leading to correction of their enophthalmos. Correction of hypoglobus (superior–inferior dimension) and overall 3D globe repositioning was clinically relevant in 28% and 62% of cases, respectively.

This study further demonstrated that in 28% of the cases, the globe position restoration was less than 1 mm in all directions. This observation raises the question of potential overtreatment of orbital floor fractures. Additionally, timing of surgery and selection of reconstruction material remain subjects of debate in the literature [5]. The decision to proceed with surgery is often based on relative indications, which can be challenging to evaluate in the immediate post-operative period due to swelling and hemorrhage. Initial diplopia often resolves spontaneously as swelling and hemorrhage subside. Consequently, various studies have suggested using surrogate parameters to predict late-onset symptoms. One such parameter is the Orbital Index, which considers the size and location of the fracture, as well as the rounding of the inferior rectus muscle [21,22]. Alternatively, a delayed surgery protocol could be considered. Except for cases with urgent indications for surgery, patients can be re-evaluated in an outpatient setting two weeks post-trauma. By this time, the swelling has typically subsided, and a new equilibrium is established, often resulting in improvement in diplopia. Dystopia can then be more accurately assessed clinically, and indications for surgery can be discussed with the patient. This delayed protocol also provides an opportunity to calculate defect size and changes in orbital volume. If surgery is indicated, a PSI can be designed and adapted for specific needs, including soft-tissue correction.

Several points of interest emerge regarding the fracture pattern. An orbital floor surface defect ratio of 50% or >2 cm^2^ is typically considered an indication for surgery. However, growing evidence suggests that it is not a reliable sole predictor for surgical intervention [23]. There is a greater chance of enophthalmos in cases with associated medial wall fractures, particularly if the inferomedial strut is fractured [8,24]. The importance of this anatomical landmark is also evident in the results of the subgroup analysis. The shift in globe position was significantly greater in the medial–lateral direction, anterior–posterior direction, and 3D Euclidean distance if the inferomedial strut was fractured. However, reconstructing this orbital buttress poses a significant challenge [25,26].

Current surgical procedures for orbital reconstruction predominantly focus on restoring the bony anatomy. To achieve this, recent developments such as virtual surgical planning using mirroring techniques, the production of PSIs and intraoperative navigation are gradually being standardized [27,28,29,30,31]. However, restoring the bony orbit is only one part of the solution. The soft tissues occupying the orbital cavity (including fat, muscles, and the neurovascular system) also impact globe position, yet these soft-tissue changes are often underestimated in contemporary protocols. Incorporating calculations of orbital volume in the pre-operative assessment and planning may improve outcomes, including globe position, by allowing for strategies such as overcorrection in PSI design. These techniques are particularly relevant for patients requiring secondary reconstruction. Although there is a growing body of literature on this topic, a consensus has yet to be reached [32,33,34].

This study is limited by its retrospective nature, relatively small cohort size, and variability in post-operative CT timing, as early imaging may be influenced by edema and transient globe displacement, potentially confounding true globe position assessment. Additionally, using DICOM images with a 2 mm slice thickness may introduce measurement errors. Therefore, it is recommended to use thin CT slices with a thickness of less than 1 mm for CT imaging, along with a standardized treatment protocol. A final limitation is that, because the applied method is independent of anatomical landmarks, clinical interpretation of globe displacement may be less straightforward without comparison to normal anatomical references, and the method is not applicable in cases of bulbar trauma with altered globe morphology.

## 5. Conclusions

In complex fracture patterns, anatomical disruption can impair the use of reference planes, which is why this study employed a landmark-independent 3D approach relying solely on the globe’s center and independent of bony references. The results demonstrate that orbital reconstruction effectively restores trauma-related globe displacements, providing a beneficial clinical outcome. This effect is most pronounced when the number of walls involved and the integrity of the inferomedial strut are carefully considered, as fractures affecting the inferomedial strut are particularly associated with greater anterior–posterior (enophthalmos), medial–lateral, and 3D (Euclidean) globe displacement. Accordingly, accurate pre-operative evaluation of fracture complexity, combined with advanced surgical planning and soft-tissue assessment, is essential to optimize outcomes and minimize residual globe displacement.

## Figures and Tables

**Table 1:** Patient characteristics, mechanism of injury, fracture patterns, and timing of surgical reconstruction.

Variable	Value				
Age	Median: 51; Mean: 52.0				
Sex	Male: n = 18 (66.7%); Female: n = 9 (33.3%)				
Mechanism of injury	Fall: n = 13 (48.1%); Sports: n = 4 (14.8%); Traffic-related: n = 3 (11.1%); Assault: n = 7 (25.9%)				
**Fracture type**	**Orbital floor**	**Medial wall**	**Lateral wall**	**Anterior maxillary sinus**	**Tripod zygoma**
n	27	16	6	11	5
%	100.0	59.3	22.2	40.7	18.5
**Time between trauma and surgery**	**Mean (days)**		**Median (days)**		**Range (days)**
Primary reconstruction	7		5		1–37
Secondary reconstruction	866		132		26–7381

**Table 2:** Quantitative analysis of three-dimensional globe position following orbital reconstruction.

Direction	Reconstruction	Unaffected Side		Z	*p*-Value
	M (±SD) (mm)	M	SD		
Anterior–Posterior	2.15 (*±1.76*)	0.82	1.01	−3.553	<0.001
Medial–Lateral	1.34 (*±1.33*)	0.48	0.64	−3.522	<0.001
Inferior–Superior	1.17 (*±1.07*)	1.05	1.06	−0.676	0.499
Euclidean distance	3.04 (*±2.12*)	1.72	1.27	−2.449	0.014

Note: Mean (M) globe shift for each direction in millimeters (mm) and corresponding standard deviation (SD). Floor reconstruction = case group (n = 29) and unaffected contralateral side = control group (n = 29). The results of the Mann–Whitney U test (assuming unequal variance) comparing the globe shift between both groups are shown (Z and *p*-value).

**Table 3:** Subgroup analysis on globe position restoration and its effect on the statistical analysis.

Subgroup	Reconstruction (ED)	*p*-Value			
	M (±SD) (mm)	AP	ML	IS	ED
**Reconstruction Material**					
Other	2.56 (*±1.83*)	0.009	0.010	0.751	0.402
PSI	3.93 (*±2.44*)	0.009	0.005	0.165	0.005
**Walls Fractured**					
1	2.39 (*±1.64*)	0.028	0.505	0.959	0.382
>1	3.28 (*±2.27*)	0.004	<0.001	0.473	0.026
**Inferomedial Strut**					
Intact	2.29 (*±1.82*)	0.064	0.223	0.448	0.614
Fractured	3.63 (*±2.22*)	<0.001	<0.001	0.128	0.002
**Clinical Pre-Operative Dystopia**					
No	2.54 (*±3.64*)	0.014	0.029	0.196	0.642
Yes	1.56 (*±2.60*)	0.022	0.066	0.026	0.006

Note: Mean (M) globe repositioning for each subgroup in 3D Euclidean distance (ED) in millimeters (mm) and corresponding standard deviation (SD). The results of the Mann–Whitney U test (assuming unequal variance) for all directions—anterior–posterior (AP), medial–lateral (ML), inferior–superior (IS), and ED—comparing the globe shift between subgroups are shown (*p*-value).

## Data Availability

The datasets generated and analysed during the current study are not publicly available due to privacy and ethical restrictions.
